# Supplementary material for: Antibiotic export by MexB multidrug efflux transporter is allosterically controlled by a MexA-OprM chaperone-like complex
Source: Nat Commun. 2020 Oct 2;11:4948. doi: 10.1038/s41467-020-18770-5 (PMC7532149; doi:10.1038/s41467-020-18770-5)
Supplement: Supplementary file 1 — Supplementary Information [file 41467_2020_18770_MOESM1_ESM.pdf]

Supplementary information

Antibiotic export by MexB multidrug efflux transporter is allosterically controlled by a MexA-OprM chaperone-like complex

Marie Glavier[1,2‖], Dhenesh Puvanendran[3‖], Dimitri Salvador[1,2‖], Marion Decossas[1,2], Gilles Phan[4], Cyril Garnier[4], Elisa Frezza[4], Quentin Cece[3], Guy Schoehn[5], Martin Picard[3], Jean-Christophe Taveau[1,2], Laetitia Daury[1,2⊥], Isabelle Broutin[4⊥*], Olivier Lambert[1,2⊥*]

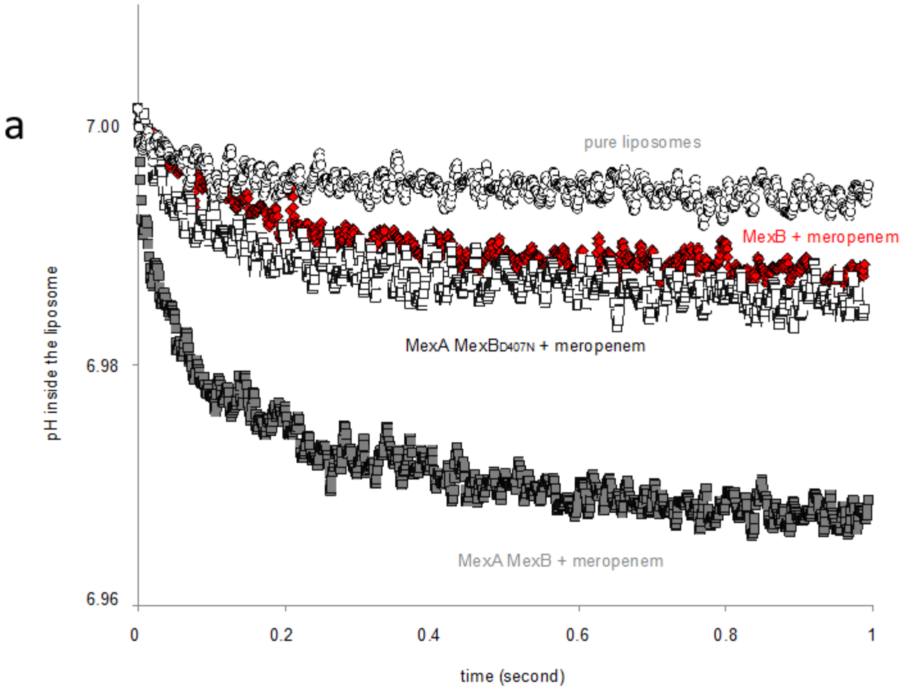

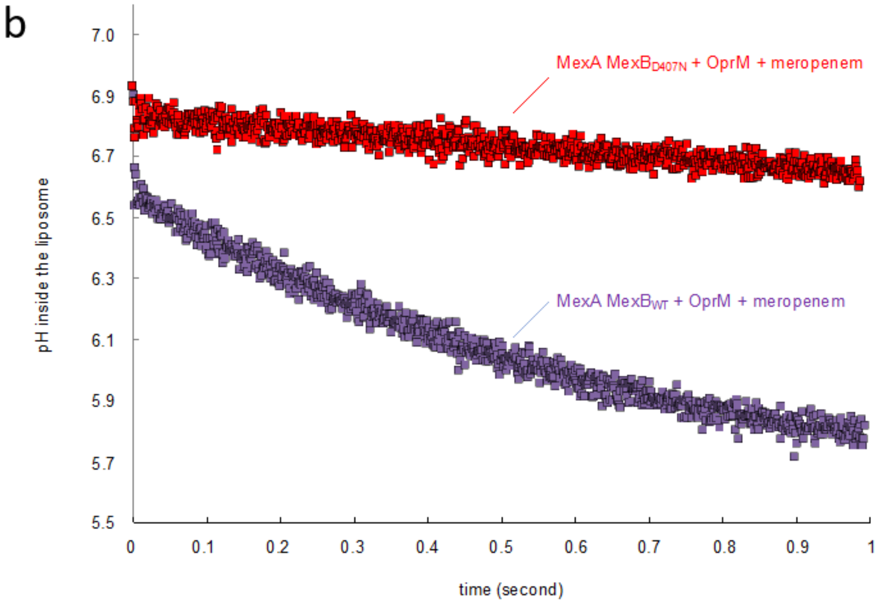

Supplementary Fig. 1: **Transport activity measurements of $MexB_{wt}$ and $MexB_{D407N}$ by monitoring kinetics of proteoliposome acidification.**

a, Wild type MexB transporter in the presence ("MexA MexB", grey squares) or in the absence ("MexB", red diamonds) of the MFP partner, or as an inactive, mutated version previously reported to be unable to counter-transport protons ("MexA $MexB_{D407N}$", white squares). Traces of $MexB_{D407N}$ (control of an inactive transport) and of MexB are similar indicating that MexB alone is inactive despite the presence of the substrate. For comparison, the presence of MexA induces a moderate activity (same trace shown in Fig. 1 orange curve). Data obtained for pure liposomes are shown as white circles. Pure liposomes and proteoliposomes were incubated for 15 minutes with meropenem and then subjected to rapid mixing with the acidification buffer (traces are the average of 5 independent reconstitution and measurements).

b, Additional controls where $MexAB_{WT}$ (purple squares) or $MexAB_{D407N}$ (red squares) were mixed with OprM proteoliposomes for 4 hours in the presence of meropenem prior to rapid mixing with the acidification buffer. Measurements performed in the presence of the inactive MexBD407N do not show any acidification in the presence of substrate and OprM. It is however to be noticed that the rate of acidification shown for the wild type proteins is different from that shown in Figure 1, most certainly because the maximal rate of acidification depends on different factors such as the yield of protein reconstituted per liposome or the efficiency of assembly between MexAB proteoliposomes and OprM proteoliposomes, hence it differs from batch to batch.

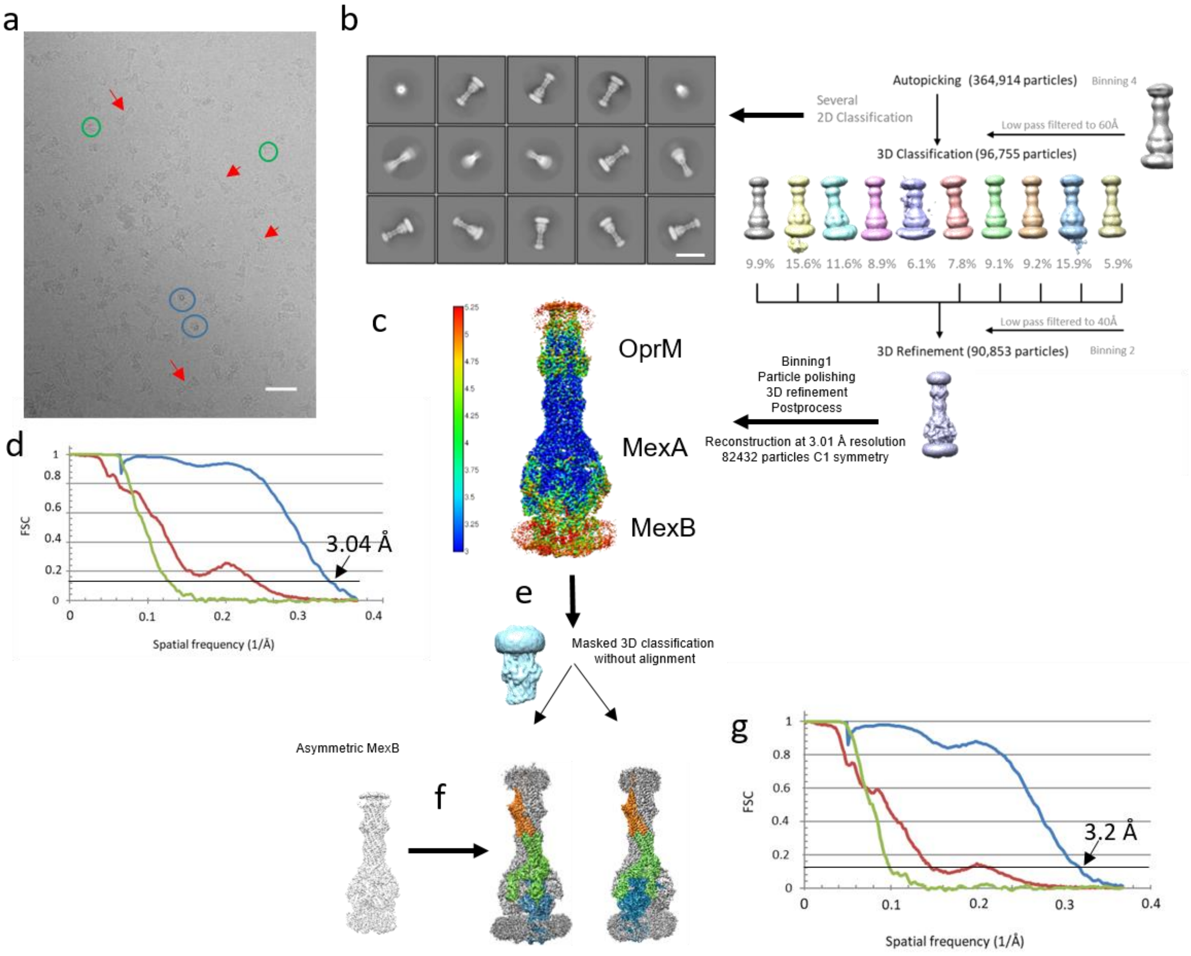

Supplementary Fig. 2: **Cryo-EM image processing procedure of tripartite complexes.**
a, Overview of cryo-EM data collection and image-processing procedure (see Methods). Micrograph representative of 3068 micrographs showing tripartite complex particles (red arrows and blue circles indicate side and top views respectively) exhibiting also views of MexB-ND (green circles) Scale bar: 50nm. **b**, Overview of image processing procedure with representative 2D class averages and 3D volumes for downscaled data. Scale bar: 30 nm. **c**, Refined 3D reconstruction coloured according to local resolution estimation (calculated using Resmap). **d**, Fourier shell correlation (FSC) curves with indicated resolution at FSC = 0.143 calculated from the 3D reconstruction in (c) before and after applying a soft mask. Masked map (blue), phase randomized masked map (green), and unmasked map (red). **e,** Mask used for sorting out two MexAB-OprM complexes presented in **f** that differ one to the other by a 60° rotation of MexB with respect to OprM. **f,** 3D Refinement using asymmetric reference determined as described in Supplementary Fig. 4. **g,** FSC curves calculated for final model. Masked map (blue), phase randomized masked map (green), and unmasked map (red).

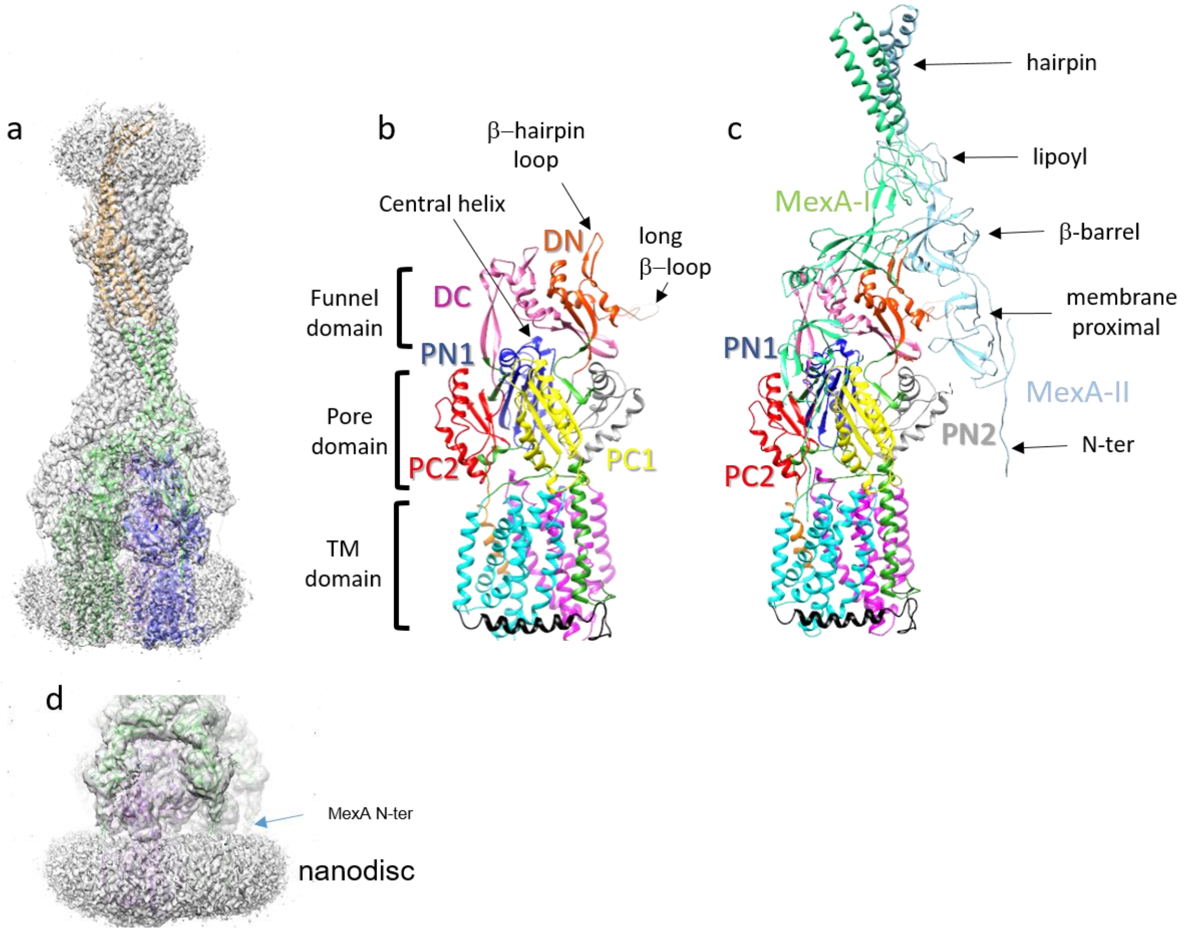

Supplementary Fig. 3: **Model of MexAB-OprM in lipid membrane.**
a, Model of the tripartite complex built from cryo-EM densities made of a OprM trimer (one monomer in orange), a trimer of MexA dimer (in cyan and green) and an asymmetric trimer of MexB. **b,c** MexB protomer (b) and MexAB complex (c). MexA dimer composed of MexA-I and MexA-II molecules interacting with MexB. MexA and MexB nomenclature is indicated. **d,** The N-terminal segments of MexA molecules are anchored into the lipid membrane of the nanodisc.

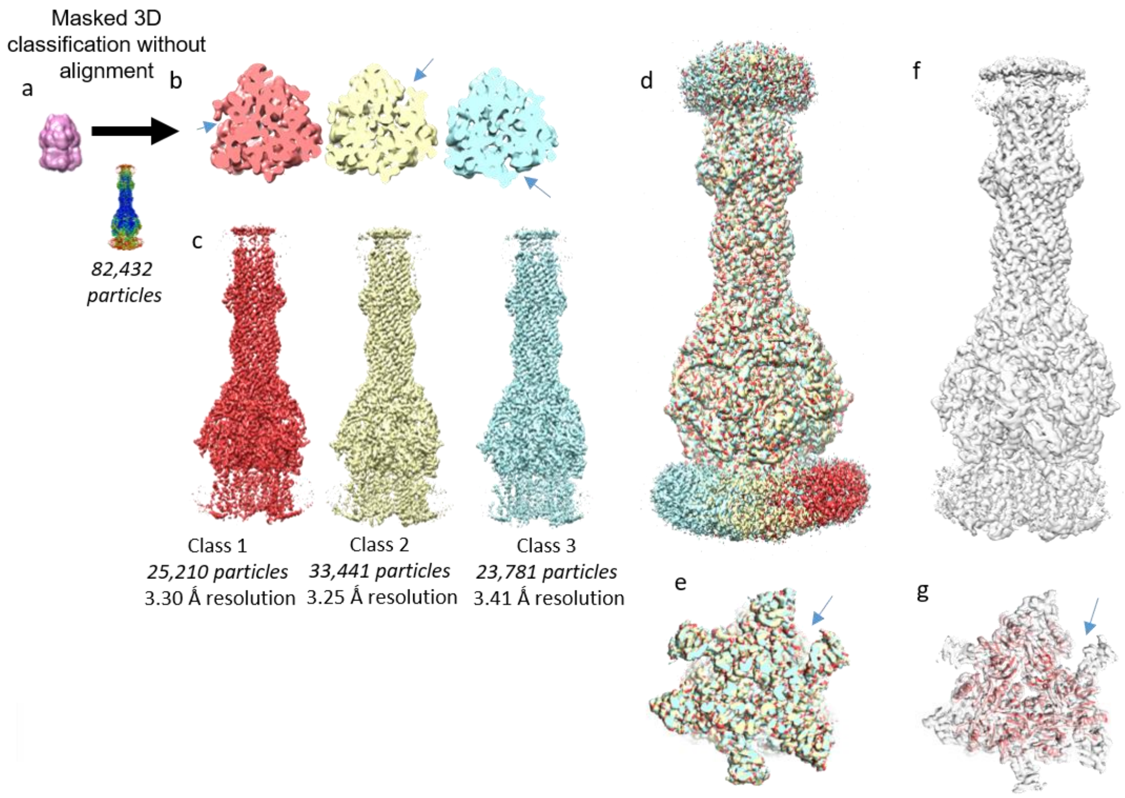

Supplementary Fig. 4: **Workflow for characterizing the asymmetric conformation of MexB within the tripartite complexes.**

a, Soft mask delineating MexB used for the masked classification without alignment procedure applied to the set of 82,432 particles. **b**, Surface representation of the three classes showing MexB in an asymmetric conformation rotated by 0°, 120 and 240° respectively. **c**, 3D reconstructions of tripartite complexes calculated from the respective classes. **d**, Superimposition of aligned volumes calculated in (c). **e,** View of the asymmetric pore domain of MexB map in (d). **f**, Average volume. **g,** View of the asymmetric pore domain of MexB map in (f). The PDB model (2V50) of crystal MexB (red) was fitted supporting the asymmetric conformation of MexB. The blue arrows indicate the pronounced cleft located in the pore domain between the T and O subunits. Note that the nanodisc is not perfectly circular as shown by the alternate colour of blue, yellow and red.

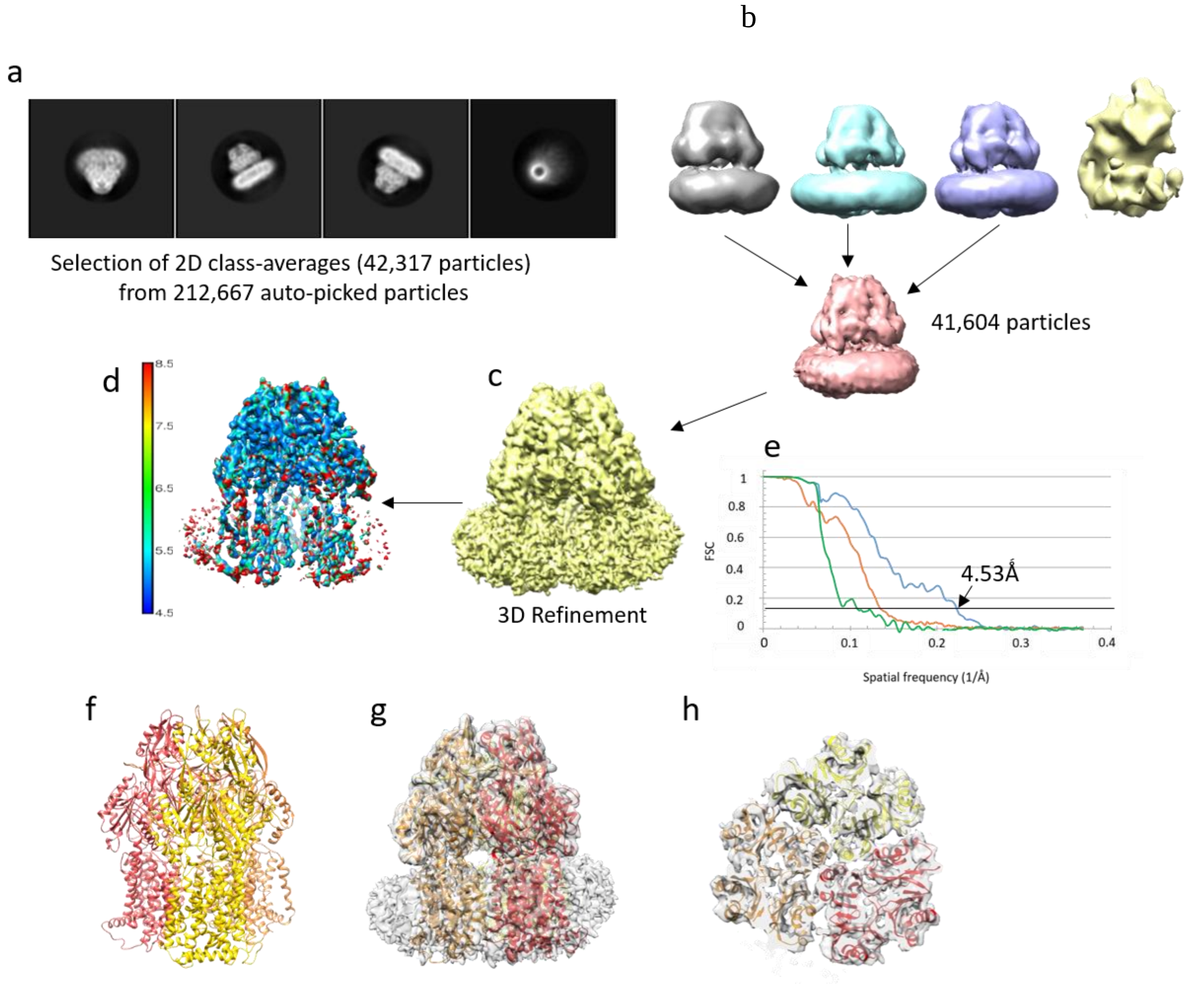

Supplementary Fig. 5: **Overview of cryo-EM image-processing of MexB-ND.**
a, Representative 2D class averages of MexB-ND particles extracted from the micrographs used for analysis of tripartite complexes (shown in Supplementary Fig. 2a). **b**, 3D classification showing the three first classes used for the final volume reconstruction. **c**, Final 3D reconstruction determined after polishing step and 3D refinement of unbinned particles. **d**, 3D map coloured according to local resolution estimation (using Resmap). A higher threshold highlights transmembrane helices. **e**, FSC curves with indicated resolution calculated for from the 3D reconstruction in (c) before and after applying a soft mask. Masked map (blue), phase randomized masked map (green), and unmasked map (red). **f**, Model of MexB-solo. **g,h,** Model fitted in EM densities of MexB-ND. Side view (g) and pore domain (h)**.**

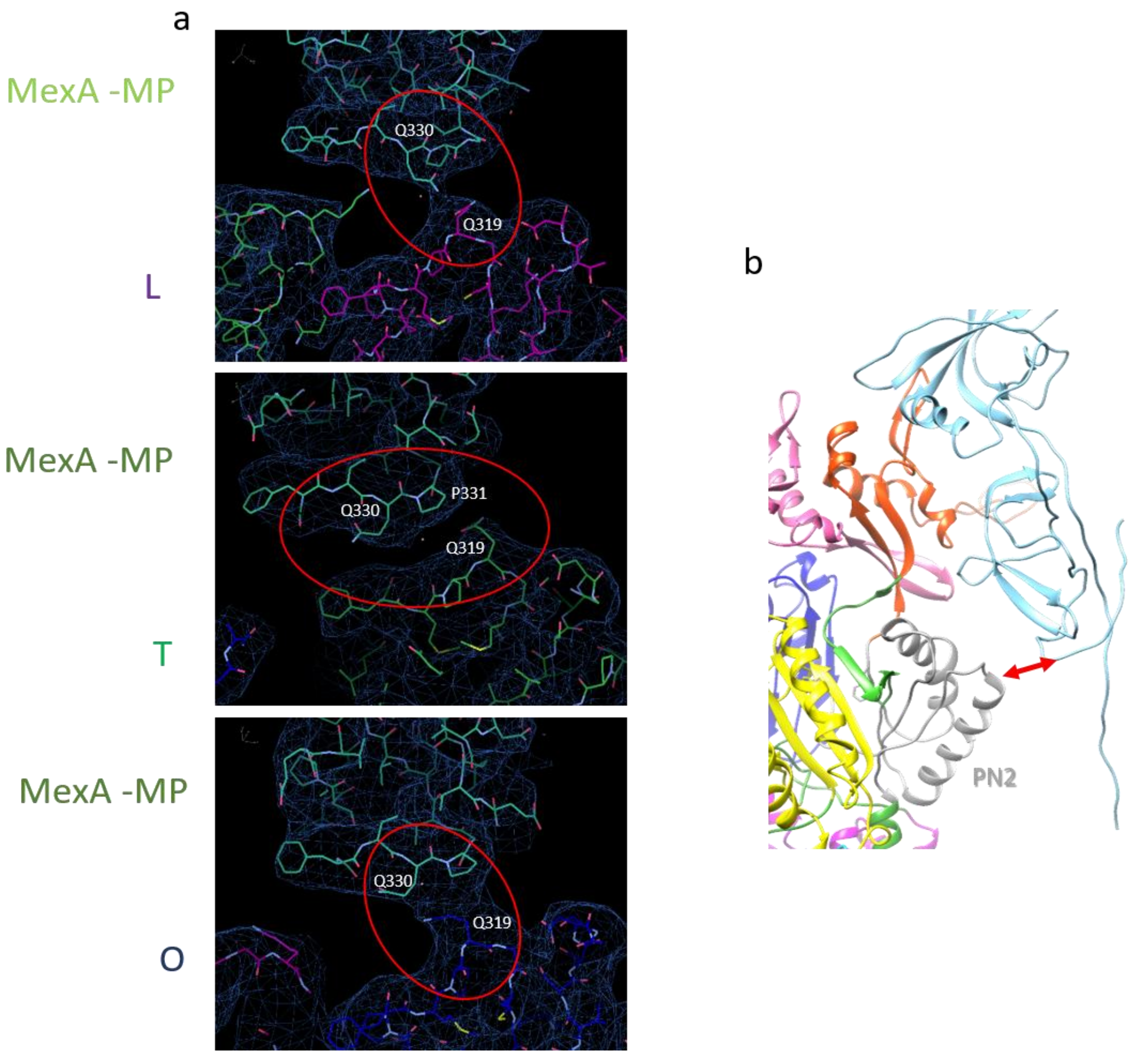

Supplementary Fig. 6: **Detailed views of contacts between the three MexA-II Membrane Proximal domains and the PN2 domains of MexB-trio protomers.**

a, EM densities of tripartite complex exhibiting MP domain contacts of MexA-II with L, T and O protomers respectively (delineated by red circles). For L and O protomers, the interface is tight involving amino acid Q330 (from MexA) and Q319 (from MexB). By contrast, for protomer T, the interface is more important involving Q330 and P331 (from MexA) and Q319 (from MexB). Note the slight displacement of densities of protomer T that shifts Q319 densities to the right creating a larger interface with its respective MP domain. **b,** Side view showing PN2 domain (grey) in contact with MexA-II MP domain (light blue). The contact is indicated by red double arrows.

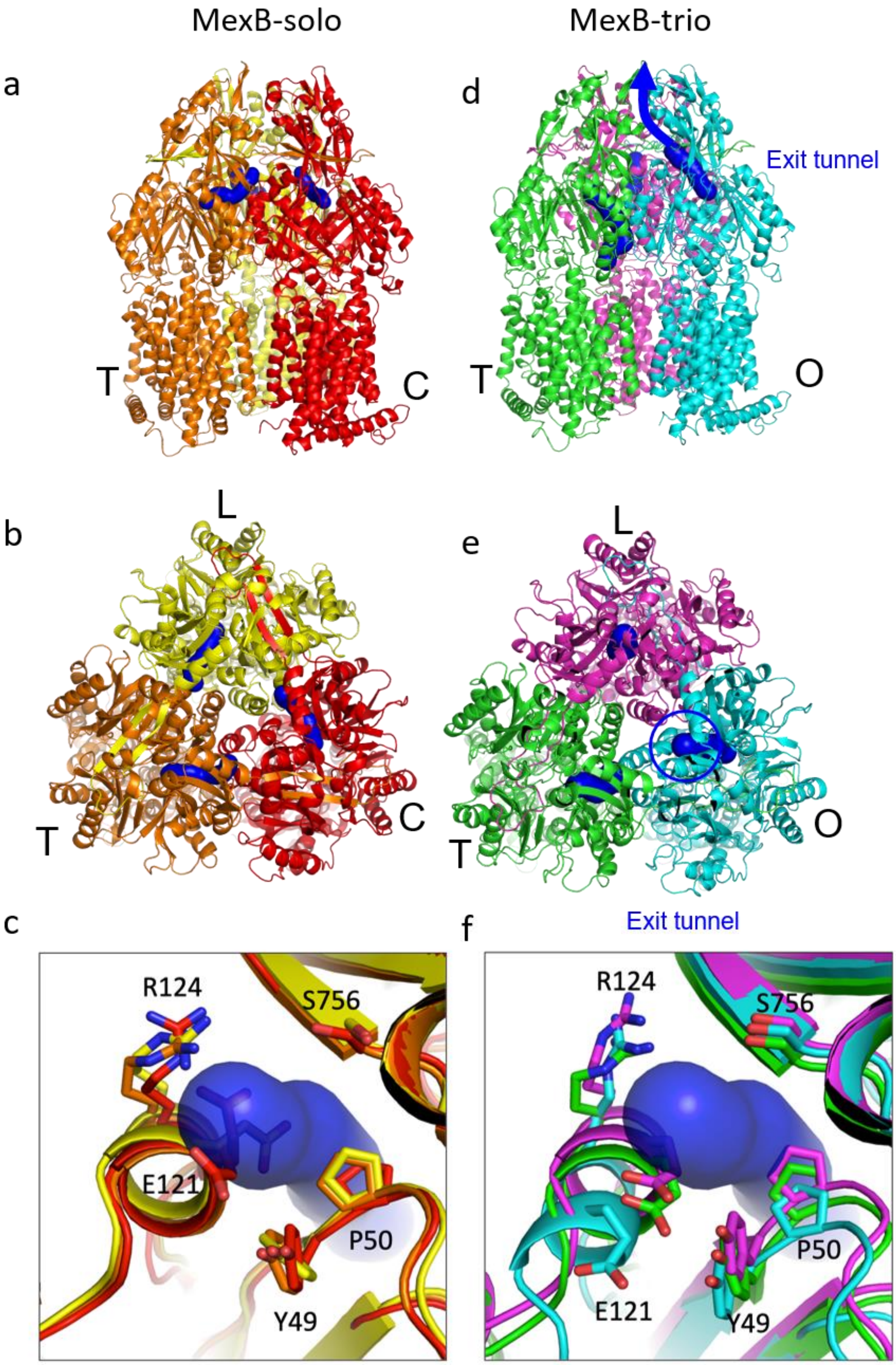

Supplementary Fig. 7: **Tunnel modelling delineating drug pathway and its exit toward funnel domain.**

Tunnels were modelled using CAVER program to delineate the pathway of drug and its exit to the funnel domain. **a, b,** Side and top views of MexB-solo. The three tunnels depicted within the L, T, C protomers have no exit. **c,** Superimposition of L, T, C protomers shows that the

tunnel gating is blocked by the helix gate (E121-R124). **d, e,** Side and top views of MexB-trio. Three tunnels are also depicted in the L, T, O protomers, with an exit path only for the protomer O (blue arrow and circle). **f,** Superimposition of L, T, O protomers shows that the tunnel exit in protomer O is mainly mediated by the shift of the helix gate (E121-R124).

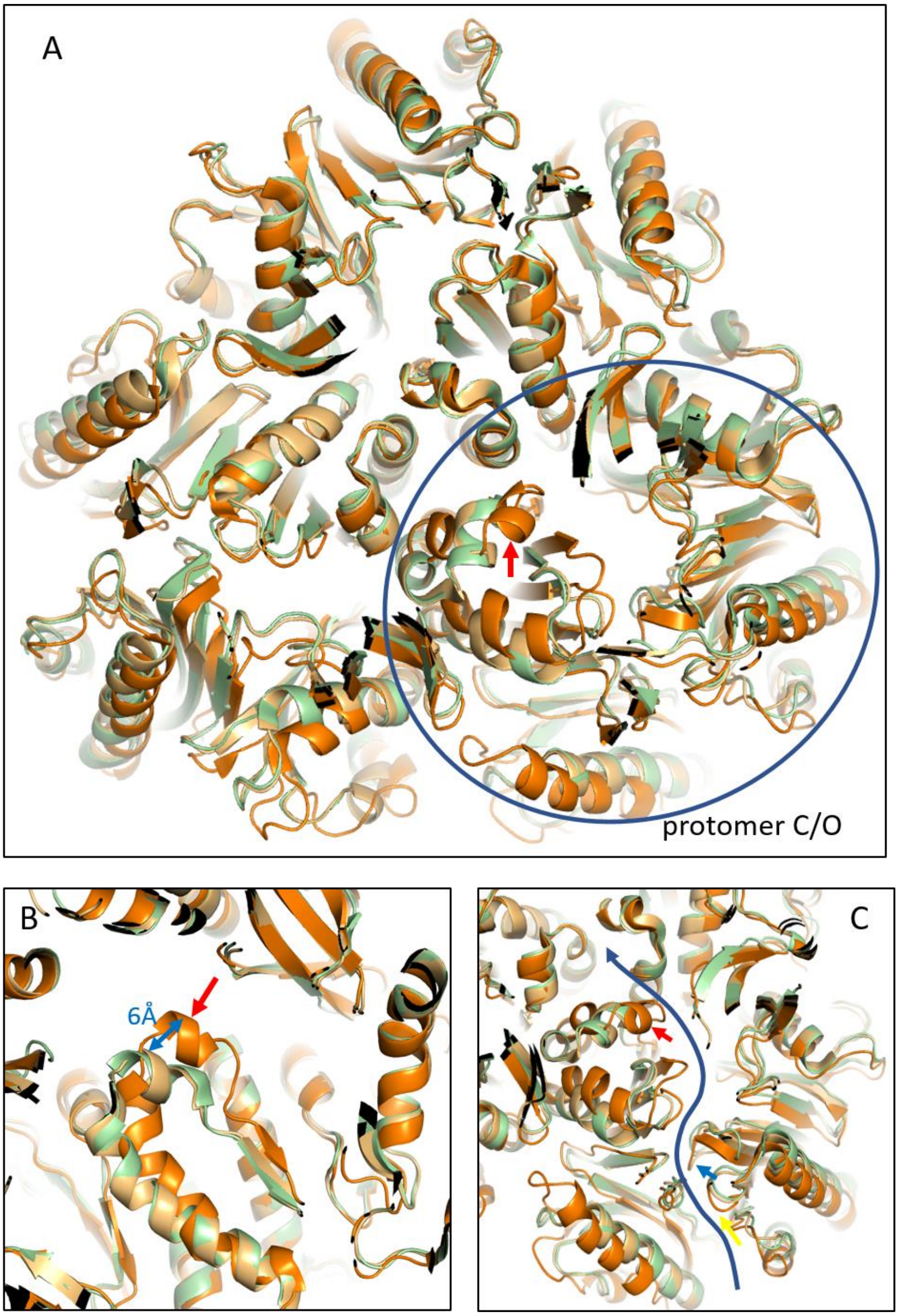

Supplementary Fig. 8: Comparison of MexB-solo with two crystal structures of MexB.

Superimposition of atomic models of MexB solo in orange, MexB without added substrate in green (PDB: 2V50) and the inhibitor-bound MexB-ABI-PP in light orange (PDB 3W9J). **a**, Top view showing structure difference in the encircled protomer C/O. The helix gate is marked with the red arrow. .**b**, Close-up view of the helix gate position in a close (red arrow) and open state corresponding to a shift of about 6 Ǻ. **c,** View delineating cavities for substrate pathway (dark blue arrow) passing successively along the gate loop (yellow arrow), the switch loop (light blue arrow) and the helix gate (red arrow).

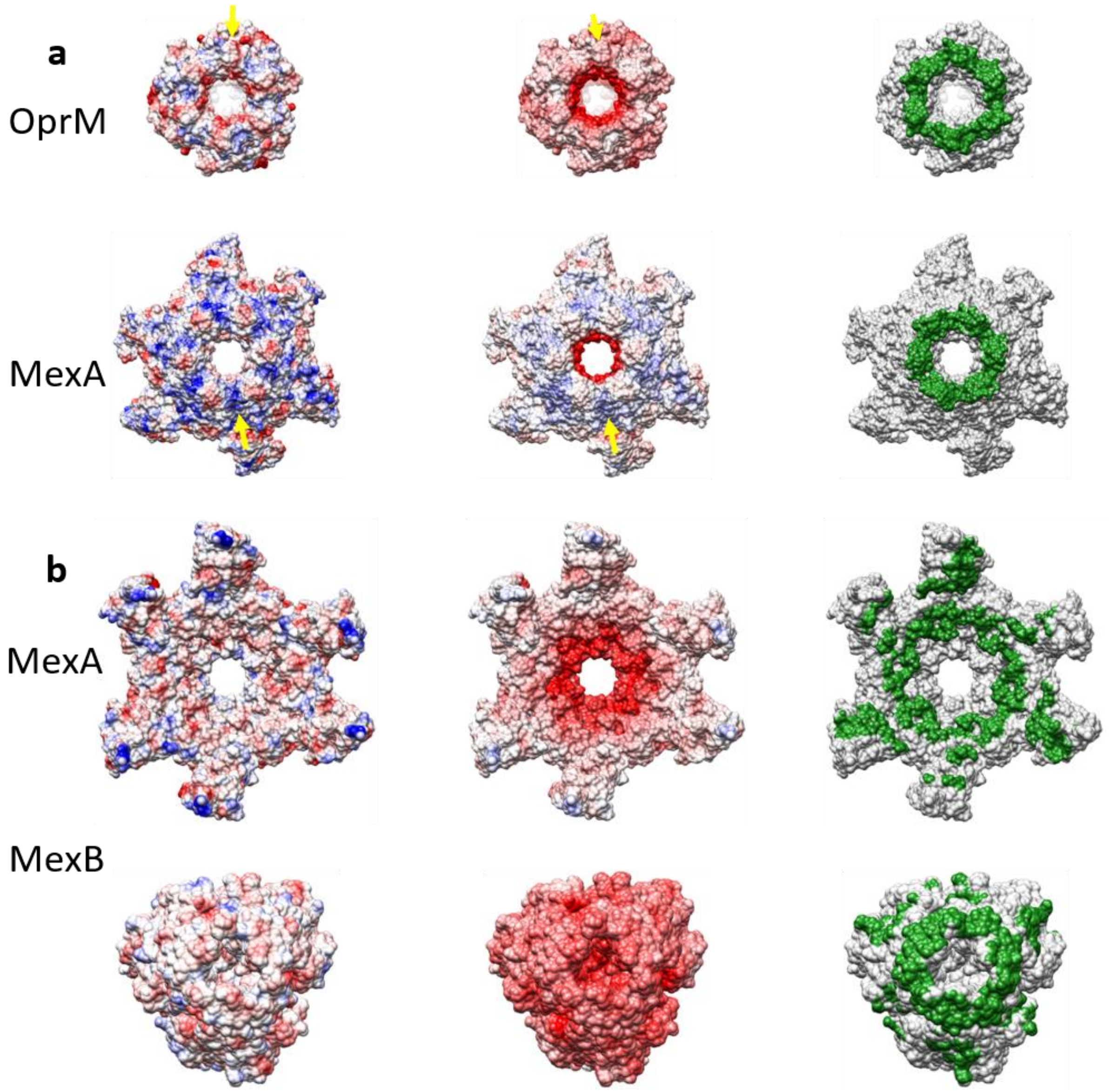

Supplementary Fig. 9: **Coulomb and electrostatic potential surfaces at the OprM-MexA and MexA-MexB interfaces.**

Two other representations of the interfaces in addition to electrostatic surfaces also shown in Fig. 4 b, d are presented to provide more details on the complementarity of OprM-MexA interface and on the respective contacts for both interfaces (green) used for energetic calculation presented in Supplementary Table 2. **a,** OprM-MexA interface. Coulomb (left), electrostatic potential surfaces (middle) generated by using Amber ff14SB force field (ε_{in} =4). The interface contacts (right) of OprM (first row) and of MexA (second row) are shown in green. Yellow arrows indicate surface complementary with negative charges of OprM facing the positive charges of MexA. **b,** MexA-MexB interface. Coulomb (left), electrostatic potential surfaces (middle) generated by using Amber ff14SB force field (ε_{in} =4) and interface contacts (right) of MexA (first row) and of MexB (second row).

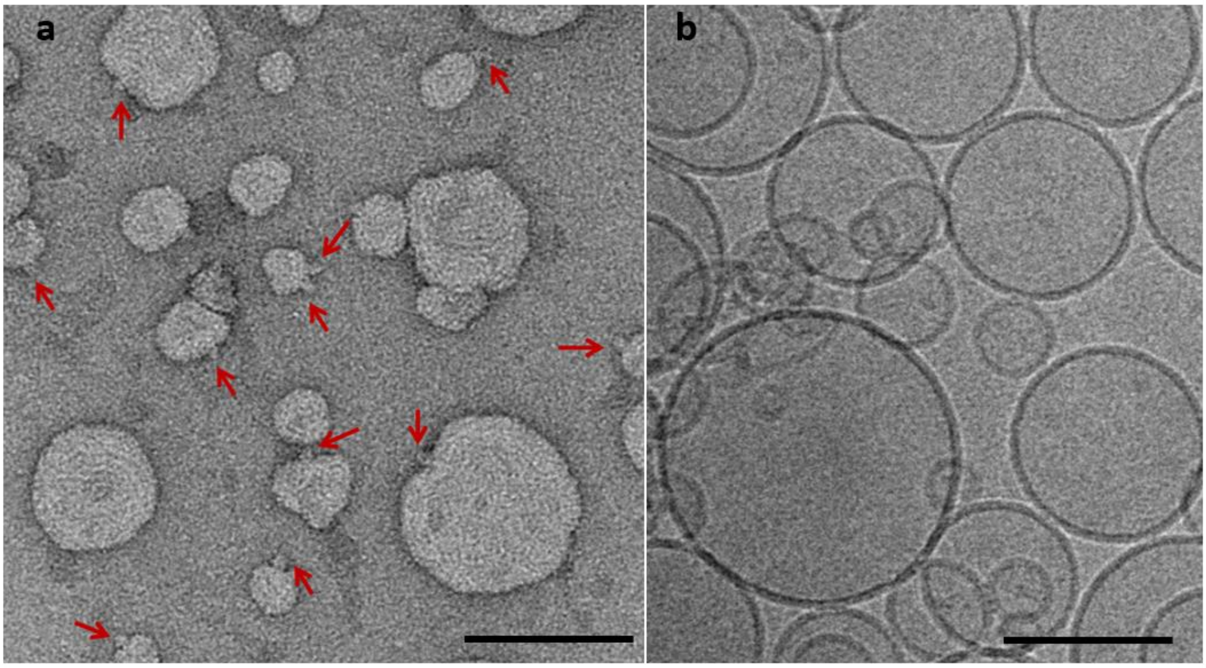

c

Proteoliposomes	*$MexB_{wt}$*	*$MexAB_{wt}$*	*$MexAB_{D407N}$*	*OprM*
Size (nm)	111.6 (± 1.1)	108.7 (± 2.2)	110.4 (± 1.9)	129.9 (± 2.4)
Polydispersity	0.2 (±0.01)	0.2 (±0.01)	0.2 (±0.02)	0.2 (±0.02)

Supplementary Fig. 10: **Proteoliposomes morphology and size measurements**
a-b, Negative-staining EM and cryoEM micrographs of MexAB proteoliposomes representative of a set of nine micrographs recorded for each experiment. Densities corresponding to MexB are visible (red arrows). Scale bars 100 nm. **c,** Average size and polydispersity measured by dynamic light scattering**.**

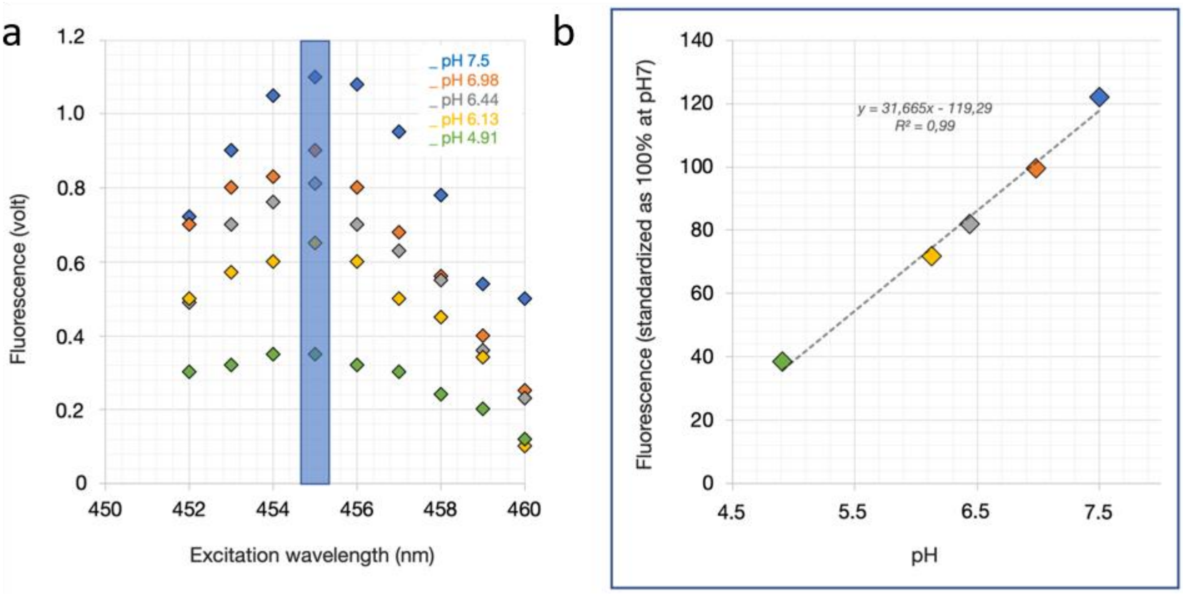

Supplementary Fig. 11: **Control of pyranine linearity over the pH range used in our study and calculation of the corresponding linear correlation**.

a, Excitation spectra performed on the pyranine-entrapped proteoliposomes used in the study. Measurements were performed on a Biologic SFM 3000 apparatus with a short pathlength optical cell (1.5 mm, "FC15" cell) and wide band emission filters centered in the region of pyranine fluorescence (MTO DA 531, obtained from MTO, Massy, France). Emitted pyranine fluorescence was measured using excitation wavelengths ranging from 452 to 460 nm on proteoliposomes resuspended in buffers with decreasing pH (see inset). Measurements were made in the presence of 10µM valinomycin and 10µM nigericin to facilitate transmembrane proton equilibration. The maximum excitation wavelength is 455 nm. **b,** Linear correlation between the percentage of fluorescence variation and the pH of the solution for an excitation wavelength of 455 nm. The calculated linear regression (Δfluorescence (%) = 31.655 x ΔpH – 119.29) was used to convert the fluorescence variations into pH variations for traces shown in Figure 1 and supplementary Fig. 1.

Supplementary Table 1: **Analysis of interface complementarity.**

Gap volume, change in accessible surface area (ΔASA) and gap index, for the OprM-MexA and MexA-MexB interfaces. Typical values of the gap index range from 1 → 5. Lower values characterize interfaces with better structural complementarity.

Interface	***ΔASA (Å²)***	***Gap volume (Å³)***	***Gap index (Å)***
OprM-MexA	2995	3276	1.1
MexA-MexB	14317	34896	2.4

Supplementary Table 2: **Energetic analysis.**

Energetic contribution to the binding free energy: change of van der Waals energy (ΔE_{vdW}), Coulomb and polar solvation energy ($\Delta E_{el}+\Delta G_{polar}$) and non-polar solvation energy (ΔG_{np}).
The sum of the three contributions provides a rough estimation of the binding free energy, without the entropic contribution that should destabilize the formation of the complex: a lower value should represent a stronger interaction between the two partners.

Interface	ΔE_{vdW}	$\Delta E_{el}+\Delta G_{polar}$	ΔG_{np}
OprM-MexA	-141	82	-33
MexA-MexB	-178	303	-156

Supplementary Table 3: Cryo-EM data collection, refinement and validation statistics.

	Tripartite complex (MexA MexB trio) EMD-10395 PDB 6TA6	Tripartite complex (MexA-OprM) EMD-10372 PDB 6TA5	MexB solo EMD-10371 PDB 6T7S
**Data collection and processing**			
Microscope	Krios @ESRF	Krios @ESRF	Krios@ESRF
Magnification	105,000	105,000	105,000
Voltage (kV)	300	300	300
Electron exposure (e^{-} Å^{-2})	42	42	42
Defocus range (µm)	-1 to -2.4	-1 to -2.4	-1 to -2.4
Pixel size (Å)	1.36	1.36	1.36
Camera	Gatan K2	Gatan K2	Gatan K2
Number of movies	3358	3358	3358
Symmetry imposed	C1	C1	C1
Initial particle images (no)	364,914	364,914	212,667
Final particle images (no)	82,432	35,819	41,604
Map resolution (Å)	3.2	3.2	4.5
FSC threshold	0.143	0.143	0.143
Map sharpening *B* factor (Å^2) as estimated by Relion		-9	-79
**Refinement**			
Model resolution (Å)	masked 3.3	masked 3.2	masked 4.6
FSC threshold	0.5	0.5	0.5
Model composition			
Non-hydrogen atoms	49550	49550	23436
Protein residues	6519	6519	3090
Ligands	0	0	0
B factors (Å^2)			
Protein	163	105	239
Ligand	-	-	-
Water	-	-	-
R.m.s deviations			
Bonds lengths (Å)	0.006	0.006	0.005
Bond angles (°)	1.217	1.243	1.260
Validation			
MolProbity score	1.66	1.61	1.80
Clashscore	5.98	4.38	7.26
Poor rotamer (%)	0.63	0.21	0.28
EMRinger	1.32	2.23	0.39
Ramachandran plot			
Favored (%)	95.29	94.18	93.97
Allowed (%)	4.60	5.61	6.03
Disallowed	0.11	0.22	0
