# Supplementary material for: Antibiotic export by MexB multidrug efflux transporter is allosterically controlled by a MexA-OprM chaperone-like complex
Source: Nat Commun. 2020 Oct 2;11:4948. doi: 10.1038/s41467-020-18770-5 (PMC7532149; doi:10.1038/s41467-020-18770-5)
Supplement: Supplementary file 3 — Description of Additional Supplementary Files [file 41467_2020_18770_MOESM3_ESM.pdf]

Description of Additional Supplementary Files

File: Supplementary Movie 1

Description: Side view of a morphing between MexB-solo and MexB–trio

File: Supplementary Movie 2

Description: Top view of a morphing between MexB-solo and MexB–trio
