# Supplementary material for: Antibiotic export by MexB multidrug efflux transporter is allosterically controlled by a MexA-OprM chaperone-like complex
Source: Nat Commun. 2020 Oct 2;11:4948. doi: 10.1038/s41467-020-18770-5 (PMC7532149; doi:10.1038/s41467-020-18770-5)
Supplement: Supplementary file 6 — Reporting Summary [file 41467_2020_18770_MOESM6_ESM.pdf]

nature research

Corresponding author(s): Olivier Lambert and Isabelle Broutin

Last updated by author(s): Sep 10, 2020

Reporting Summary

Nature Research wishes to improve the reproducibility of the work that we publish. This form provides structure for consistency and transparency in reporting. For further information on Nature Research policies, see Authors & Referees and the Editorial Policy Checklist.

Statistics

For all statistical analyses, confirm that the following items are present in the figure legend, table legend, main text, or Methods section.

n/a	Confirmed	
☐	☒	The exact sample size (*n*) for each experimental group/condition, given as a discrete number and unit of measurement
☐	☒	A statement on whether measurements were taken from distinct samples or whether the same sample was measured repeatedly
☒	☐	The statistical test(s) used AND whether they are one- or two-sided<br>*Only common tests should be described solely by name; describe more complex techniques in the Methods section.*
☒	☐	A description of all covariates tested
☒	☐	A description of any assumptions or corrections, such as tests of normality and adjustment for multiple comparisons
☐	☒	A full description of the statistical parameters including central tendency (e.g. means) or other basic estimates (e.g. regression coefficient) AND variation (e.g. standard deviation) or associated estimates of uncertainty (e.g. confidence intervals)
☒	☐	For null hypothesis testing, the test statistic (e.g. *F*, *t*, *r*) with confidence intervals, effect sizes, degrees of freedom and *P* value noted<br>*Give P values as exact values whenever suitable.*
☒	☐	For Bayesian analysis, information on the choice of priors and Markov chain Monte Carlo settings
☒	☐	For hierarchical and complex designs, identification of the appropriate level for tests and full reporting of outcomes
☒	☐	Estimates of effect sizes (e.g. Cohen's *d*, Pearson's *r*), indicating how they were calculated

Our web collection on statistics for biologists contains articles on many of the points above.

Software and code

Policy information about availability of computer code

Data collection	Data collection on Titan krios using EPU 1.11
Data analysis	MotionCor2.1, GCTF 1.06, Relion 2.1 and Relion 3.0, ResMap 1.1.4, Chimera 1.10.2, phenix 1.14, phenix 1.17, Coot 0.8.9.1, Caver analyst 2.0 , Delphi8.0

For manuscripts utilizing custom algorithms or software that are central to the research but not yet described in published literature, software must be made available to editors/reviewers. We strongly encourage code deposition in a community repository (e.g. GitHub). See the Nature Research guidelines for submitting code & software for further information.

Data

Policy information about availability of data

All manuscripts must include a data availability statement. This statement should provide the following information, where applicable:

- Accession codes, unique identifiers, or web links for publicly available datasets
- A list of figures that have associated raw data
- A description of any restrictions on data availability

Atomic coordinates of the conformations have been deposited to the Protein Data Bank with accession number 6T7S for conformations LTC and 6TA5,6TA6 for LTO . The cryo-EM density maps have been deposited in the Electron Microscopy Data Bank with accession number 10371 for conformations LTC and 10372,10395 for LTO.PDB: 3W9J for MexB, PDB:2V4D and PDB:5NG5 for MexA, PDB:3D5K for OprM were used for starting model building

Field-specific reporting

Please select the one below that is the best fit for your research. If you are not sure, read the appropriate sections before making your selection.

☒ Life sciences ☐ Behavioural & social sciences ☐ Ecological, evolutionary & environmental sciences

For a reference copy of the document with all sections, see nature.com/documents/nr-reporting-summary-flat.pdf

Life sciences study design

All studies must disclose on these points even when the disclosure is negative.

Sample size	Sample size was determined based on prior published data from similar experiments (Daury et al, Nat commun, 7 10731 (2016);Tsutsumi et al, Nat commun, 10 1520 (2019))
Data exclusions	According to cryoEM principle, particles having poor qualities were excluded after rounds of 2D and 3D classification allowing to get high resolution cryo EM structure of the class of interest. For transport activity studies, no data were excluded
Replication	All biochemical experiments were repeated three times and are all reproducible
Randomization	All experiments were performed with clonal populations
Blinding	No blinding was performed as the acquisition and analysis require human intervention

Reporting for specific materials, systems and methods

We require information from authors about some types of materials, experimental systems and methods used in many studies. Here, indicate whether each material, system or method listed is relevant to your study. If you are not sure if a list item applies to your research, read the appropriate section before selecting a response.

Materials & experimental systems

n/a	Involved in the study
☒	☐ Antibodies
☒	☐ Eukaryotic cell lines
☒	☐ Palaeontology
☒	☐ Animals and other organisms
☒	☐ Human research participants
☒	☐ Clinical data

Methods

n/a	Involved in the study
☒	☐ ChIP-seq
☒	☐ Flow cytometry
☒	☐ MRI-based neuroimaging
